# Book Reviews

**Published:** 1904-06

**Authors:** 


### BOOK REVIEW.

Anaesthesia in Dental Surgery, by Thos. D. Luke, M. B., F. R. C. S. E. New York, Rebman Co., 10 W. 23rd St., 1903. Price, $1.50.

In this little volume will be found a large amount of interesting data regarding the anesthetics and anesthetic combinations used in dental surgery.

The ten chapters take up the History of Anaesthesia, Nitrous Oxide, Gas and Oxygen, Gas and Ether, Ethyl Chloride and Ether, Anaesthetics in Special Cases, Local Anaesthesia, Chloroform, Accidents.

This book should be in the hands of every dentist who makes a practice of giving anaesthetics.

---

Eye Strain in Health and Disease. With special reference to the amelioration or cure of chronic nervous derangements without the aid of drugs. By Ambrose L. Ranney, A. M., M. D. Illustrated, royal octavo. 325 pages. Extra cloth. Beveled edges. Net, $2.00. The F. A. Davis Co., 1914 Cherry St., Philadelphia, publishers.

While this work naturally is one which must be of peculiar interest to eye specialists, the subject matter cannot fail to be of value to the dentist upon whose eyes there is such a constant strain.

It is probably the most complete and scientific embodiment published of the view that eye strain is a very important factor in the causation of many chronic diseases.

We can recommend it as a good book for the dentist to study carefully.

